# Cytoprotective Effects of β-Melanocortin in the Rat Gastrointestinal Tract

**DOI:** 10.3390/molecules171011680

**Published:** 2012-10-01

**Authors:** Mirna Bradamante, Petra Turčić, Nikola Štambuk, Paško Konjevoda, Gorana Aralica, Ivan Alerić, Ana Kozmar

**Affiliations:** 1 Department of Dermatology and Venerology, University Hospital Center Zagreb, Šalata 4, 10000 Zagreb, Croatia; Email: mirna.bradamante@kbc-zagreb.hr; 2 Department of Pharmacology, Faculty of Pharmacy and Biochemistry, University of Zagreb, Domagojeva 2, 10000 Zagreb, Croatia; Email: pturcic@pharma.hr; 3 Ruđer Bošković Institute, Bijenička cesta 54, 10002 Zagreb, Croatia; 4 University Hospital Dubrava, Avenija Gojka Šuška 6, 10000 Zagreb, Croatia; Email: garalica@kbd.hr; 5 Department of Pulmology, University Hospital Canter Zagreb, Kišpatićeva 12, 10000 Zagreb, Croatia; Email: i.aleric@inet.hr; 6 Department of Laboratory Diagnostics, University Hospital Center Zagreb, Kišpatićeva 12, 10000 Zagreb, Croatia; Email: akozmar@kbc-zagreb.hr

**Keywords:** β-melanocortin, cytoprotection, gastritis, colitis, TNBS, hepatoprotection

## Abstract

Recently discovered anti-inflammatory and immunomodulatory properties of melanocortin peptides led to the conclusion that they might serve as new anti-inflammatory therapeutics. The purpose of this work was to examine the effectiveness of β-melanocortin (β-MSH) in two experimental models: ethanol-induced gastric lesions and TNBS (2,4,6-trinitrobenzenesulfonic acid)-induced colitis in male Wistar rats. Three progressive doses of β-MSH were used: 0.125, 0.250 and 0.500 mg/kg. Our results suggest that β-MSH acts as a protective substance in the gastric lesions model, which can be seen as a statistically significant reduction of hemorrhagic lesions at all three doses, compared to the control group. The most efficient dose was 0.250 mg/kg. Statistically significant reduction in mucosal surface affected by necrosis and the reduction of overall degree of inflammation in the colitis model indicates an anti-inflammatory effect of β-MSH at a dose of 0.250 mg/kg. The results justify further research on β-MSH peptide and its derivates in the inflammatory gastrointestinal diseases, and point out the possibility of using β-MSH in studies of digestive system pharmacology.

## 1. Introduction

Gastrointestinal inflammation represents a major public health problem in all parts of the World. Effectively dealing with the problem of gastrointestinal inflammation is difficult due to the complicated issues of unknown etiology, inefficient treatment and the side effects of existing anti-inflammatory drugs. The melanocortins could be possible therapeutic agents for the treatment of gastrointestinal inflammation, including inflammatory bowel disease (IBD) [1,2,3]. They represent peptides derived from the pro-opiomelanocortin (POMC) hormone by proteolytic cleavage and include α-MSH, β-MSH and γ-MSH [2,3]. Melanocortins are multifunctional neuropeptides that exert cytoprotective, anti-inflammatory and immunosuppressive effects on many different organs and tissues, as shown by numerous preclinical studies [1,2,3,4,5,6,7]. Their effects are elicited through the tissue-specific expression of five melanocortin receptors (MC1R-MC5R) [3,4]. The most investigated melanocortin hormone, in the context of tissue inflammation and cytoprotection, is α-MSH [2,3,7]. Its protective effects in the gastrointestinal tract were investigated and confirmed in experimental models of gastritis, colitis and acetaminophen-induced hepatitis [5,6,8,9,10,11]. Contrary to numerous studies on the effects of α-MSH, relatively little is known about the effects of β-MSH. 

The sequence of β-MSH in vertebrates is not evolutionarily conserved. β-MSH in humans has 22 amino acids (AEKKDEGPYRMEHFRWGSPPKD) whereas in most mammals, this molecule is shorter and contains 18 amino acids [12]. β-MSH is an agonist for MC1R, MC3R and MC4R, with the highest affinity for the MC4R. In the central nervous system (CNS) melanocortin peptides are agonists of MC3R and MC4R [13,14,15,16,17,18]. The central mode of β-MSH action involves the regulation of feeding, metabolism, preservation of body weight, *i.e.*, regulation of energy homeostasis through the well characterized neuronal pathway—central melanocortin system [13,17,18]. The central melanocortin system also influences inflammation in the central nervous system [13,14,15,16,17,18] and β-MSH, like ACTH or α-MSH, blocks the activation of transcription factor NF-κB and stops the secretion of cytokines, chemokines and the expression of adhesion molecules [14,15,16]. Peripheral mode of melanocortin action is mostly due to MC1R and MC5R [1,13,17]. Through the MC1 receptor β-MSH is involved in the regulation of pigmentation and inflammation, however, its peripheral cytoprotective and anti-inflammatory effect was not much investigated [5]. For now, the only known anti-inflammatory and cytoprotective effect of β-MSH in the gastrointestinal tract was studied on the model of acetaminophen-induced hepatitis, where it showed stronger effects than α-MSH [5]. Consequently, we further investigated the cytoprotective and anti-inflammatory effects of β-MSH in the rat gastrointestinal tract by using standard experimatal models of gastritis and colitis.

## 2. Results and Discussion

### 2.1. Cytoprotective Effect of β-MSH on Ethanol-Induced Gastritis

**Figure 1:**
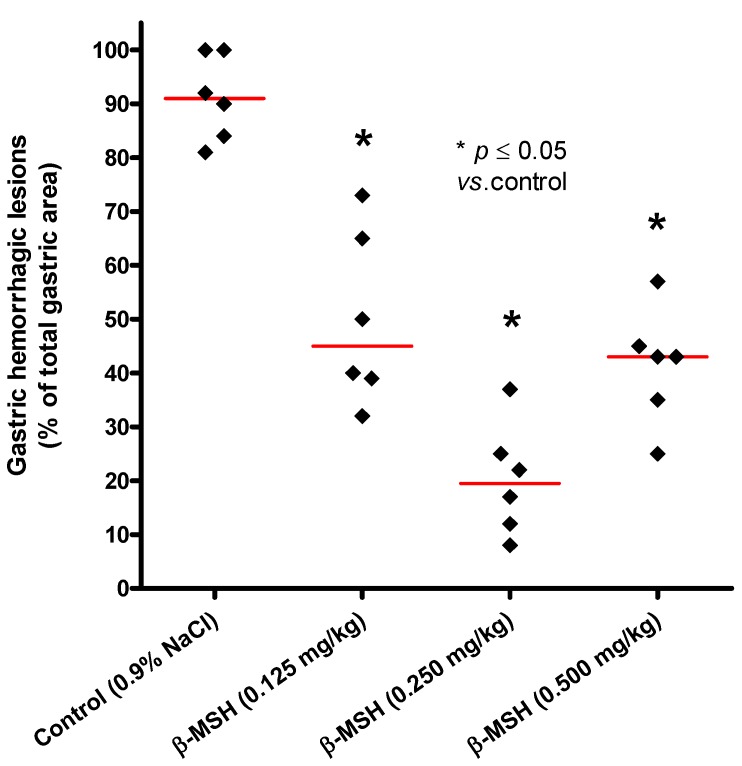
Area of hemorrhagic lesions as a percentage of total gastric area for individual animals in each experimental group. Diamonds represent individual values, and red lines denote median. *p* value is a result of comparison with the control group (Steel’s test).

**Figure 2:**
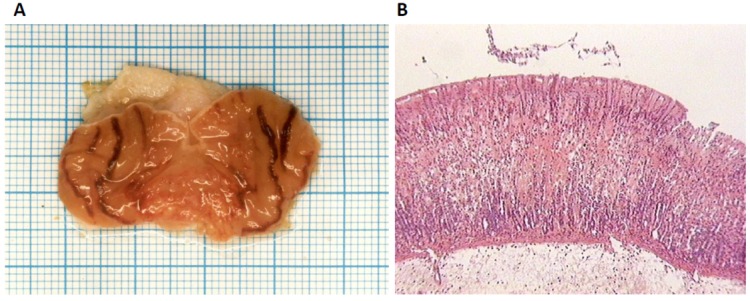
(**A**) Hemorrhagic lesions area of the ethanol-induced gastritis in male Wistar rat treated with β-MSH (0.250 mg/kg b.w.). (**B**) Histological view of the gastric mucosal damage in rats using a light microscopy (hematoxilin and eosin staining, magnification ×50). Presence of edematousmucosainfiltratedwith inflammatory cells and extravasated erythrocytes.

The effect of β-MSH was first estimated on the ethanol-induced gastric lesions model, which is a reliable screening model for the evaluation of gastric cytoprotection [8,11,19]. Our results showed that β-MSH acts cytoprotective on the model of ethanol-induced gastritis at all three applied doses (0.125, 0.250 and 0.500 mg/kg), which can be seen in a statistically significant reduction of hemorrhagic lesions compared to the control group (*p* ≤ 0.05, Table 1, Figure 1 and Figure 2).

**Table 1:** Descriptive statistics (area of hemorrhagic lesions as a percentage of total gastric area) for experimental groups in ethanol-induced gastritis. *p* value is a result of comparison with the control group (Steel’s test).

Substance	i.p. dose	n	Mean	SD	Median	*p* value
Control	0.9% NaCl	6	91.20	7.91	91.0	
β-MSH	0.125 mg/kg	6	49.80	16.12	45.0	0.011
β-MSH	0.250 mg/kg	6	20.20	10.34	19.5	0.011
β-MSH	0.500 mg/kg	6	41.00	11.90	43.0	0.017

The strongest cytoprotective effect was observed when the middle-dose of 0.250 mg/kg β-MSH was applied. In this group hemorrhagic gastric lesions amounted to 20.2% of the total gastric mucosa area (Steel test, *p* = 0.011 *vs.* control; Table 1 and Figure 1). Control (0.9% NaCl) exhibited a significantly higher percentage of the gastric lesions (median area of lesions 91.0%). β-MSH doses of 0.125 and 0.500 mg/kg were also cytoprotective and the percentages of hemorrhagic lesions in those groups were 19.5% and 43.0%, respectively (Table 1 and Figure 1). The protective effect of β-MSH has the U-shaped dose-response (Figure 1), a common finding in the field of peptide research [5,20]. The increase in dose (from 0.250 to 0.500 mg/kg) was followed by diminishing of the protective effects (Figure 1). This finding is usually explained as a result of non-specific binding of tested substance to other receptors and molecules [5,20].

### 2.2. Cytoprotective and Antiinflammatory Effect of β-MSH on TNBS-Induced Colitis

Cytoprotective and anti-inflammatory effects of β-MSH were also investigated in the model of rat experimental colitis induced by intrarectal application of TNBS/ethanol. The experimental model of TNBS-induced colitis is a highly reproducible, dose-dependent, inexpensive, simple and popular animal model of human inflammatory bowel disease [21,22]. This robust model is a standard screening model for cytoprotective agents and drugs [21,22]. β-MSH doses of 0.125, 0.250 and 0.500 mg/kg were used for the treatment. Cytoprotective and anti-inflammatory effects of the peptide were evaluated by comparing the colon lesions in β-MSH treated groups to the lesions in control (untreated) animals. In TNBS-induced colitis, all of the histological sections examined showed a full thickness necrosis of the mucosa with a moderate mixed inflammatory infiltrate of the underlying layers (submucosa, muscularis propria and serosa). Since the microscopic pictures of all the specimens examined were identical, the estimation of the β-MSH efficacy was not based on microscopic but rather on macroscopic characteristics. Colonic necrosis and macroscopic inflammation score were the parameters used for the evaluation of the protective effects of β-MSH on TNBS-induced colitis [9,11,21,22].

**Figure 3:**
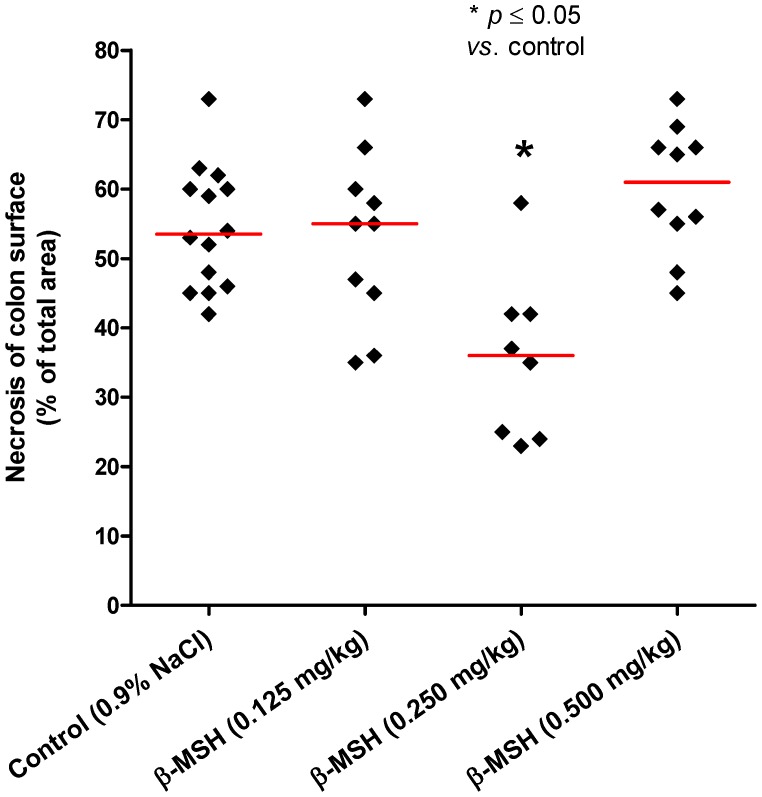
The area of colonic necrosis as a percentage of total colon area for individual animals in each experimental group. Diamonds represent individual values, and red lines denote median. *p* value is a result of comparison with the control group (Steel’s test).

**Figure 4:**
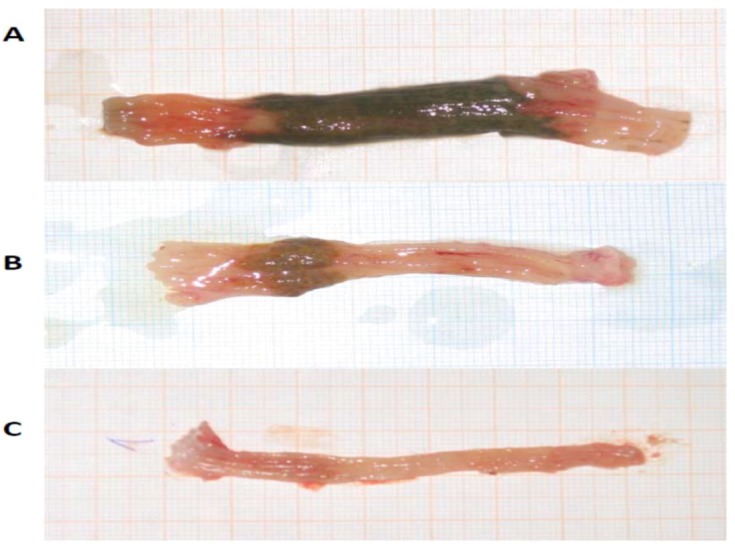
(**A**) Area of colon mucosal lesions induced by TNBS, with necrotic areas (72%). Control animal treated with 0.9% NaCl. (**B**) Area of colon mucosal lesions induced by TNBS, with necrotic areas (23%). Animal treated with β-MSH (0.250 mg/kg b.w.). (**C**) Native control, colon without necrotic areas.

The area of colonic necrosis was expressed as a percentage of total colon area, and the results of β-MSH treated animals were then compared to the results of controls (0.9% NaCl) (Table 2, Figure 3 and Figure 4). The doses of 0.125 and 0.500 mg/kg β-MSH did not show any effect on the reduction of colonic necrosis after intrarectal application of TNBS/ethanol (Steel test, *p* > 0.05, Table 2 and Figure 3). However, the middle-dose of β-MSH (0.250 mg/kg) significantly reduced the colonic necrosis (Steel’s test, *p* = 0.004, Table 2 and Figure 3). Median values of the colon necrotic area measured in the control group (53.5%) significantly dropped in the group of rats treated with 0.250 mg/kg β-MSH (36.0%, Table 2).

**Table 2:** Descriptive statistics (area of colonic necrosis as a percentage of total colon area) in experimental model of colitis. *p* value is a result of comparison with the control group (Steel’s test).

Substance	i.p. dose	n	Mean	SD	Median	*p* value
Control	0.9% NaCl	14	54.40	8.82	53.5	
β-MSH	0.125 mg/kg	10	53.00	12.30	55.0	0.995
β-MSH	0.250 mg/kg	8	35.75	11.90	36.0	0.004
β-MSH	0.500 mg/kg	10	60.00	9.23	61.0	0.308

Similar results were observed with respect to the macroscopic inflammation score (Table 3). In control animals the median value of the colon inflammation score was 3, however, in animals treated with 0.250 mg/kg β-MSH the mean value of the score dropped significantly (median value 2, *p* = 0.013, Table 3). The results indicate that the middle-dose of β-MSH (0.250 mg/kg) significantly reduced the macroscopic inflammation of the colon in TNBS-induced colitis. The lower β-MSH dose (0.125 mg/kg) and a higher β-MSH dose (0.500 mg/kg) did not reduce macroscopic inflammation of the colon (*p* > 0.05, Table 3). The observed U-shaped relationship between dose and the effects of β-MSH on TNBS-induced colitis (Figure 3 and Table 2) is in line with previously reported effects of this peptide on experimental gastritis. U-shaped dose-response curve is characteristic for the peptides, including other melanocortins, e.g., similar results were detected for α-MSH in experimental colitis, gastritis and hepatitis [5,6,8,9,10,11].

**Table 3:** Descriptive statistics (macroscopic inflammation score, 0–3 scale) in experimental model of colitis. *p* value is a result of comparison with the control group (Steel’s test).

Substance	i.p. dose	n	Individual scores	Mean	SD	Median	*p* value
Control	0.9% NaCl	14	3, 2, 3, 3, 2, 3, 3, 3, 3, 3, 3, 3, 3, 3	2.86	0.36	3.0	
β-MSH	0.125 mg/kg	10	3, 2, 2, 3, 2, 3, 3, 3, 2, 3	2.60	0.52	3.0	0.376
β-MSH	0.250 mg/kg	8	2, 2, 3, 1, 2, 2, 3, 2	2.13	0.64	2.0	0.013
β-MSH	0.500 mg/kg	10	3, 3, 3, 2, 3, 3, 3, 3, 2, 3	2.80	0.42	3.0	0.972

### 2.3. Evaluation of Systemic and Local Inflammation in TNBS-Induced Colitis

The most prominent pro-inflammatory mediators in TNBS-induced colitis are prostaglandins, tromboxane, tumor necrosis factor α (TNF-α), interleukin 1 (IL-1), interleukin 6 (IL-6) and others [20]. Anti-inflammatory effect of melanocortins is thought to be predominantly mediated by cellular factor NF-κB inhibition. NF-κB regulates the transcription of more than 150 genes that are included in production of cytokines, chemokines and other pro-inflammatory agents [23,24]. However, determination of pro-inflammatory cytokines in the intestinal wall was disabled due to extensive mucosal necrosis. Therefore, the concentrations of TNF-α and IL-6 were measured in the plasma of experimental animals. Plasma levels of pro-inflammatory cytokines TNF-α and IL-6 were below the detection limits of the tests (<12.5 pg/mL for TNF-α and <62.5 pg/mL for IL-6) confirming that, in this model of acute inflammation, there was no systemic inflammatory response [25]. Higher values of pro-inflammatory cytokines, particularly TNF-α are the characteristics of a chronic TNBS model [25]. Our results are in line with previous observations that the evaluation of the colonic mucosal necrosis and macroscopic inflammation score represent reliable parameters for the evaluation of protective effects in TNBS-induced colitis [9,11,19,21,22]. 

**Figure 5:**
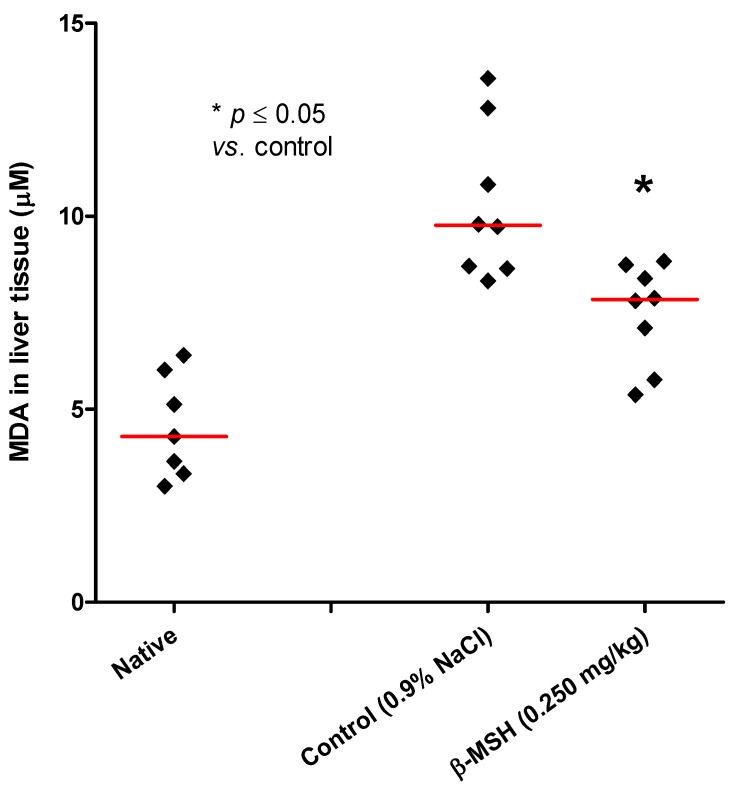
Level of liver tissue malondialdehyde (MDA) in native animals, control (0.9% NaCl) and β-MSH (0.250 mg/kg b.w.) treated groups. Diamonds represent individual values, and red lines denote median. *p* value is a result of comparison with the control group (Mann-Whitney test).

TNBS-induced colitis is primarily characterized by lesions of the colon and histological picture of acute inflammatory reactions [19,20]. However, endogenous substances leaked from damaged colon in male Wistar rats with TNBS induced colitis activate Kupffer cells, leading to the down-regulation of hepatic cytochrome P450 enzymes, mild inflammatory conditions and oxidative stress [26]. This side-effect of TNBS on hepatic P450 enzymes in acute experimental colitis is without liver injury [26]. We confirmed the results of Masubuchi *et al.* [26] by the histopathological estimation of liver damage. Liver tissue was formalin-fixed, paraffin-embedded. Routine hemalaun-eosin slides were analyzed. In all experimental groups with TNBS-induced colitis, liver architecture was preserved and all liver tissue components were in normal ranges, without any pathological changes. 

Malonyldialdehyde (MDA) levels in the liver of the controls and β-MSH treated groups were analyzed considering the fact that hepatic cytochrome P450 enzymes down-regulation and oxidative stress are found in TNBS-induced colitis [26]. MDA is an aldehydic product of lipid peroxidation that has been the most used as a marker of the oxidative stress [27]. In the Wistar rats ß-MSH significantly reduced liver levels of MDA from 9.76 μM (interquartile range 8.66–12.31) in the control group to the level of 7.84 μM (interquartile range 6.10–8.65) in the group treated with 0.250 mg/kg ß-MSH (Figure 5, Mann-Whitney test, *p* = 0.007). This result confirms the involvement of melanocortin system/β-MSH in the regulation of the resistance to oxidative stress [28]. These effects are thought to be MC1 receptor mediated [28]. We did not observe significant difference in the plasma levels of MDA between the control group and experimental group of animals treated with β-MSH (0.250 mg/kg). This result is consistent with the fact that the experimental model of TNBS-induced colitis used in our experiment is primarily characterized by lesions of the colon and histological picture of acute inflammatory reactions, without systemic inflammation [19,20]. 

**Figure 6:**
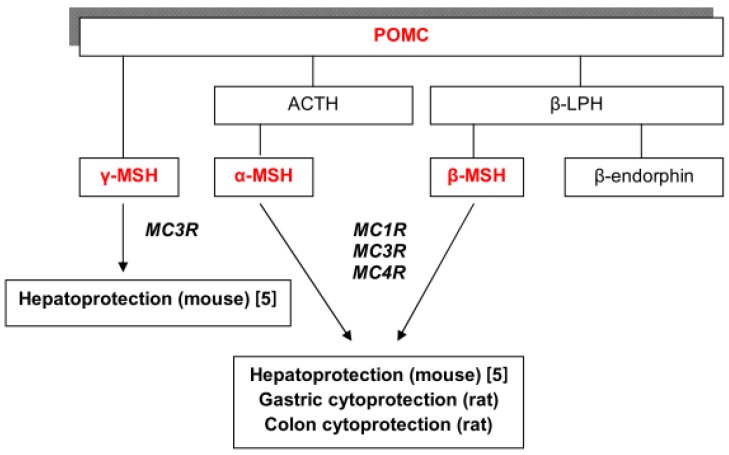
Melanocortin peptides and related receptors involved in the gastrointestinal cytoprotection.

It is believed that α-MSH achieves its protective effects in the colitis model via the MC1 receptors (Figure 6) [3]. Synthetic agonists of MC1 receptors, known as MS05 and MS09 also show anti-inflammatory effects [29]. MC1 receptor is a potential target for developing new anti-inflammatory therapy in inflammatory bowel disease and specific allele mutations of this receptor may be associated with a higher risk of developing IBD [29]. Our results indicate that intestinal inflammation could be also modulated via melanocortin receptor agonist β-MSH. β-MSH is a MC1, MC3 and MC4 receptors agonist but it shows the highest affinity for the MC4 receptors, exerting the effects through MC1, MC3 and MC4 receptors (Figure 6) [29]. Known effects of β-MSH in the gastrointestinal tract are regulation of feeding, body weight and metabolism, primarily through the MC4 receptor [13,17,18]. β-MSH has much stronger hepatoprotective effects then α-MSH, using criteria of potency and efficacy [5]. The same is valid for the β-MSH cytoprotection in the gut, since optimal protective dose in experimental colitis and gastritis model is 0.250 mg/kg for β-MSH (Table 1, Table 2 and Table 3) and 1 mg/kg for α-MSH [8,9,11]. The role of melanocortin receptors and their agonists in the regulation of immune homeostasis in the intestine is poorly researched. The use of selective antagonists could identify the role of individual melanocortins and their receptor subtypes (MC1R-MC5R) [5]. This study represents a potentially useful source of knowledge about the regulation of inflammation in the gut and liver.

## 3. Experimental

### 3.1. Animals

Male Wistar rats, bred at the Department of Pharmacology, School of Medicine, Zagreb, Croatia, weighing between 200 and 250 g, were used for this study. Animals were kept in a room with constant temperature (21 ± 1 °C) and a dark-light cycle (12 h/12 h), housed in cages, maximum six rats per cage. They were fed with standard laboratory rodent pellets (4RF21, Mucedola, Milan, Italy) and given water *ad libitum.* Each experimental group contained eight rats. 

### 3.2. Materials

1. Synthetic β-melanocortin (β-MSH, AEKKDEGPYRMEHFRWGSPPKD, GenScript, Piscataway, NJ, USA, purity > 95%) was used at three doses: 0.125 mg, 0.250 mg and 0.500 mg per kilogram of body weight
2. 2,4,6-trinitrobenzenesulphonic acid (TNBS, Sigma-Aldrich Inc., St. Louis, MO, USA) dissolved in 40% ethanol
3. 96% ethanol (Kemika, Zagreb, Croatia)
4. 0.9% NaCl

### 3.3. Treatment Regimen

Experiments were performed according to the ILAR Guide for the Care and Use of Laboratory Animals, Council Directive 86/609/EEC, and Croatian Animal Protection Act (Official Gazette 135/06). Animals were fasting for 24 h prior to inducing gastritis or colitis [19]. Tested substances were given intraperitoneally one hour prior to the gastritis or colitis provocation.

Gastritis was induced with intragastrical application of 1 mL of 96% ethanol. One hour later animals were sacrificed; stomach was removed and opened along the greater curvature [8,11]. 

Colitis was induced with TNBS solution (30 mg/kg TNBS + 40% ethanol) [19,21,22]. After light anesthesia rats were injected with 1 mL of TNBS/ethanol into the anus via a rubber rectal catheter, the solution was retained in the gut cavity at a depth of 8 cm. Rats were sacrificed 72 h after TNBS application, when maximal extent of colonic damage was expected and the last 10 cm of colon was examined. Serum was obtained for biochemical analysis.

### 3.4. Evaluation of Mucosal Lesions Damage

Images of hemorrhagic gastric lesions and images of colonic necrosis were taken with a digital camera (JVC) in order to evaluate the extent of mucosal damage [8,11,21,30]. The public domain processing program, ImageJ was then used to analyze the images and measure total area (mm^2^) of the diseased phenotype [30]. Mucosal injury degree was defined as the sum of the areas of all lesions and expressed as percentage (%) in relation to the total mucosal area [20]:


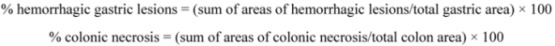


### 3.5. Macroscopic and Histopathological Estimation of Colon

Immediately after dissection the colon was visually assessed for inflammation according to the following macroscopic inflammation score: (0) no visible damage; (1) slight inflammation, slight redness (hyperemia), villi visible at 15× magnification; (2) intermediate inflammation, discontinuous hyperemia, intermediate redness of villi; (3) intensive inflammation, intensive hyperemia, intensive redness of villi [20]. Sections of the colon were fixed in 4% phosphate buffered formalin, embedded in paraffin, cut into slices and stained with the hemalaun-eosin method. Sections were examined by using a light microscope at 100× magnification. 

### 3.6. Evaluation of Systemic Inflammation

Plasma levels of pro-inflammatory cytokines tumor necrosis factor-α (TNF-α) and interleukin-6 (IL-6) were measured using ELISA kits (R&D Systems, Minneapolis, MN, USA). A monoclonal antibody specific for rat IL-6 or TNF-α had been pre-coated onto a microplate. Standard, control and plasma samples were pipetted into the wells and any rat IL-6 or TNF-α present was bound by the immobilized antibody. After 2 h incubation unbound substances were discarded, and wells were rinsed. Then, enzyme-linked polyclonal antibody specific for rat IL-6 or TNF-α was added to the wells, the system was incubated for 2 h and rinsed again. The substrate solution (hydrogen peroxide + tetramethylbenzidine) was added, incubated for 30 min, then protected from any light. The addition of the stop solution (hydrochloric acid solution) resulted in a color change of blue to yellow. The absorbance was measured at 450 nm with a microplate reader (Dynatech Laboratories Inc., MR5000, Chantilly, VA, USA). Concentrations of TNF-α and IL-6 were expressed in pg/mL (minimum detectable concentration was 12.5 pg/mL for TNF-α and 62.5 pg/mL for IL-6, as indicated by the manufacturer).

Malondialdehyde (MDA) levels in plasma and liver were determined by MDA-TBARS assay, a colorimetric determination by the thiobarbituric acid reactive substances (TBARS) [27]. Chemicals and reagents were as follows: BHT [2,6 *di*-*ter*-*butyl*-4-methylphenol, ≥99.0% (GC); (Sigma-Aldrich Inc., St. Louis, MO, USA)], TCA (trichloroacetic acid, ≥98%; Sigma-Aldrich Inc.), TBA (thiobarbituric acid, ≥98%; Sigma-Aldrich Inc.), ethanol 98% (Merck, Darmstadt, Germany), HCl (37%; puriss. p.a. Sigma-Aldrich Inc.) and KCl (≥99%; Sigma-Aldrich Inc.). The 0.2 g of liver tissue was used to obtain 10% homogenates in 0.15 M KCl that were further treated with 25 µL 0.2% BHT (in 98% ethanol) as an antioxidant. The homogenates with BHT were transferred into Eppendorf tubes, centrifuged (Hettich Universal 32 R, Tuttlingen, Germany) at 18,890 g for 20 min, and followed by transfer of the supernatants into Nunc CryotubeE, successive additions of 5% aqueous TCA in proportion 1:4 and recentrifuged (Hettich Universal 32 R) at 1780 g for 15 min. TCA was used for protein precipitation because of its low toxicity. The aliquots of 500 µL of deproteinized supernatants were transferred into the Kartell microtubes, and 500 µL TBA 0.375% in 0.25 M HCl was added. It was heated at 100 °C for 15 min, followed by cooling the samples to room temperature and measuring MDA levels by UV–VIS spectrophotometry (HPV-220, Iskra, Kranj, Slovenia) using 1-cm absorption cell. The concentration of MDA was calculated by reading the absorbance at 532 nm using a molar extinction coefficient of e = 1.56 × 10^5^ M⁄cm. Concentrations of MDA in samples are expressed in µM. During the procedure (until heating), samples were kept on ice.

### 3.7. Statistical Analysis

Statistical analysis was made by using KyPlot Software version 4 and GraphPad Prism version 5 [31,32]. Kruskall-Wallis, Steel test, and Mann-Whitney test were used to determine the differences between effects of applied β-MSH doses and control group (0.9% NaCl). *p* values ≤ 0.05 were considered as statistically significant.

## 4. Conclusions

1. The optimal protective dose ofβ-MSH in experimental colitis and gastritis model is 0.250mg/kg b.w.
2. β-MSH acts as a cytoprotective agent in themodelof ethanol-induced gastritis, which can be seenas astatisticallysignificant reduction ofhemorrhagiclesions when compared to the controlgroup.
3. In the model of TNBS-induced colitis β-MSH significantly reduced necrosis of the colonic mucosa and overalldegree ofinflammation, when comparedto the control group.
4. The presentedresultsjustifyfurther research on β-MSH peptideanditsderivativesin the inflammatorygastrointestinal diseases, and point to the possibility of using them in studies of digestive system pharmacotherapy.
